# Pirtobrutinib: a promising therapy for overcoming the resistance of ibrutinib in mantle cell lymphoma

**DOI:** 10.1097/MS9.0000000000002045

**Published:** 2024-04-15

**Authors:** Sania Kaneez Fatima, Sara Khan, Zaib Un Nisa Mughal, Hussain Sohail Rangwala, Burhanuddin Sohail Rangwala, Mohammad Arham Siddiq, Mirha Ali, Asma Ahmed Farah

**Affiliations:** a Department of Medicine, Jinnah Sindh Medical University; b Department of Medicine, East Africa University, Boosaaso, Somalia

## Introduction

Mantle cell lymphoma (MCL) is a rare kind of B-cell non-Hodgkin lymphoma, which develops when a B lymphocyte in the ‘mantle zone’, or outer edge of a lymph node follicle, undergoes a malignant transformation. It frequently begins with unregulated B lymphocyte transformation-related lymph node growth before spreading to other organs, such as the bone marrow, liver, and gastrointestinal system. MCL tumor cells, which commonly co-express CD5, consist of small-sized to medium-sized lymphoid cells with irregular nuclei that are typically monomorphic and lack pseudo-follicles, para-immunoblasts, and centroblasts^1^. MCL is usually sporadic, but it is possible that some families have a higher prevalence of this disease. One case of mantle cell lymphoma occurs annually in every 200 000 people. About 5% of non-Hodgkin’s lymphomas are MCL^2^.

Middle-aged and elderly individuals are most commonly affected by this condition, with an average age of 50–70 years. Additionally, it affects men at least twice as often as it affects women. Most patients already had advanced disease by the time they first arrived. Intestinal involvement may be present in some patients with lymphomatous polyposis, which accounts for a large proportion of cases^3^. Extranodal involvement frequently affects the peripheral blood, intestine, and bone marrow.

Additionally, the patient developed painless lymph node swelling in the neck, armpits, and groin. There was persistent fatigue, fever, nocturnal dyspnea, unexplained weight loss, and itchy skin^4^.

Patient age, disease stage, and overall health status influence lymphoma treatment. Numerous chemotherapeutic medications have been suggested for the treatment of MCL. Rituximab, doxorubicin (adriamycin), vincristine sulfate (oncovin), cyclophosphamide, hydroxyl-daunomycin, Lenalidomide, and Ibrutinib are among those that are employed^5^. Dexamethasone and prednisone are the two primary corticosteroids used to treat lymphomas. Patients with MCL may also benefit from the use of kinase inhibitors, selective inhibitors of antiapoptotic protein B-cell lymphoma 2 (Bcl-2 inhibitors), and proteasome inhibitors^6^. Radiation therapy is one of the most effective treatments for mantle cell lymphomas. High-energy X-rays are used in radiation treatment to treat MCL^7^. Chemotherapy is still the cornerstone of first-line treatment for people with MCL, and it frequently follows stem cell transplantation^8^. The status of newer medications, including Bruton tyrosine kinase (BTK) inhibitors and chimeric antigen receptor T-cell therapy, has advanced to second-line and third-line treatment options. All three BTK inhibitors that have been approved to date (ibrutinib, acalabrutinib, and zanubrutinib) function by irreversibly binding to an active site of the protein known as the C481 binding site. However, the covalent bond formed by this binding leads to the emergence of resistance mutations at the C481 site, which can render these drugs ineffective^9,10^.

To treat adult individuals with relapsing or refractory MCL, the FDA has granted approval for the drug pirtobrutinib (Jaypirca) because of its favorable response rate in patients who have undergone a minimum of two rounds of systematic therapy along with a BTK inhibitor^11^. Pirtobrutinib inhibits BTK function by binding to it noncovalently. Pirtobrutinib’s inhibitory action is sustained despite the mutations in the region, such as Cys481, are expressed, distinguishing it from the other types of BTK inhibitors that bound irreversibly to the BTK active site^12^.

BRUIN clinical trials are evaluating the effectiveness of pirtobrutinib in relapsed and refractory B-cell malignancies in adults. They began in 2020 with a phase 1/2 study conducted on 323 patients across seven dose ranges (25–300 mg QD), which concluded that the drug is safe and effective for a variety of B-cell malignancies, including patients who had been administered covalent BTK inhibitors. Phase 2 dosage recommendations are 200 mg per day^13^. It was determined that the phase 1/2 data could be useful in selecting the first-line BTK inhibitor therapy in relapsed MCL in 2022 after 60 more patients had well-tolerated and satisfactory results. However, many patients require additional treatments. The medication is associated with several side effects such as black, foul-smelling stools, gum bleeding, blood in the urine and stools, body pain, coughing, chest tightness, congestion in the ears, fever, chills, loss of ability to speak, skin pallor, red pinpoint spots on the skin, nasal congestion, sore throat, swelling, breathing difficulties, unexpected bruising, bleeding, fatigue, and an unusual lack of strength^14^. A Phase 3 open-label, randomized trial was launched in 2022 to compare the effectiveness of pirtobrutinib monotherapy and monotherapy of covalent BTK inhibitors (such as ibrutinib, acalabrutinib, or zanubrutinib) in those patients who had been treated with BTK inhibitors in the past. This study is currently ongoing, with the aim of achieving improved outcomes. It is expected that the newly developed medication will overcome resistance to earlier covalent BTK inhibitors^15^.

As of 16 July 2021, 618 patients in the BRUIN Phase I/II study were treated. Fatigue (23%), diarrhea (19%), neutropenia (18%), and contusion (17%) were the most frequent treatment-emergent adverse events, regardless of attribution or grade, seen in ≥15% of patients. The most common emergent adverse event (grade ≥3) was neutropenia (8%). Bruising (22%), all grade 1 or 2 events, was the only adverse event of special interest that occurred in ≥15% of patients, regardless of attribution. Six patients (1%) discontinued pirtobrutinib owing to treatment-related adverse events^16^.

Of the 100 efficacy-evaluable patients with BTK inhibitor-pretreated MCL, the overall response rate (ORR) per Lugano classification was 51% (95% CI: 41–61), including 25 patients with complete response and 26 with partial response. Among the 11 patients with BTK inhibitor-naive MCL, the ORR was 82% (95% CI: 48–98), including two patients with complete response and seven with partial response. With a median follow-up of 8 months (range, 1.0–27.9 months) for responding patients, the median duration of response was 18 months (95% CI: 4.6–not estimable). At the data cut on 16 July 2021, 60% (36 of 60) of the responses were ongoing^16^.

Based on the robust activity of pirtobrutinib in the phase I/II trial in heavily pretreated MCL patients (median lines of prior therapy 3 [Interquartile range (IQR): 2–4]), it is hypothesized that pirtobrutinib will be more efficacious than the currently approved covalent BTK inhibitors (ibrutinib, acalabrutinib, or zanubrutinib) when administered as first-line BTK inhibitor therapy in relapsed MCL.

## Conclusion

A remarkably specific non-covalent agent ‘Pirtobrutinib’, that restrains the activity of BTK, has been shown to exhibit sustained inhibitory effects even when Cys481 mutations are present. Conversely, covalent BTK inhibitors such as ibrutinib bind directly to Cys481 in the BTK active site. Pirtobrutinib on the other hand, binds noncovalently to the site while efficiently inhibiting BTK activity. The oral route of administration provides significant advantages in maintaining continuous BTK inhibition over the course of the dosing regimen, irrespective of the inherent rate of turnover of BTK. Pirtobrutinib has shown encouraging efficacy in patients with B-cell malignancies who had received prior therapy, such as covalent BTKi, in these cases.

Through phase 1/2 clinical research of the BRUIN trial, pirtobrutinib has been subjected to comprehensive evaluation for the treatment of MCL and other B-cell malignancies. The effectiveness of this treatment has been shown to maintain a constant level of efficacy irrespective of the occurrence or nonoccurrence of genetic mutations. The findings revealed that pirtobrutinib showed favorable efficacy in MCL, with a poor prognosis and extensive pretreatment after several rounds of therapies, including covalent BTK inhibitors. Pirtobrutinib was easily tolerated and safe, and it was effective over a wide range of dosages. The phase 1/2 BRUIN trial substantiated the endorsement of pirtobrutinib. This was specifically observed in patients diagnosed with relapsed or refractory mantle cell lymphoma who had previously undergone treatment with covalent BTK inhibitors, as they exhibited an overall favorable response rate.

Hence, pirtobrutinib may be regarded as a viable and effective therapeutic approach for managing and regulating MCL and other malignancies of B-cell origin.

## Ethical approval

Ethics approval was not required for this editorial.

## Consent

Informed consent was not required for this editorial.

## Sources of funding

The authors received no extramural funding for the study.

## Author contribution

The conceptualization was done by H.H. and B.S.R. The literature and drafting of the manuscript were conducted by H.H., S.M.F.Z., S.M.H., A.S.J., H.S.R., M.A., and A.A.F. The editing and supervision were performed by B.S.R. All authors have read and agreed to the final version of the manuscript.

## Conflicts of interest disclosure

The authors declare no potential conflicts of interest concerning the research, authorship, and/or publication of this article.

## Research registration unique identifying number (UIN)

Not applicable.

## Guarantor

Not applicable.

## Data availability statement

Not applicable.

## Provenance and peer review

Not applicable.

## References

[1] Swerdlow SH Campo E Pileri SA . The 2016 revision of the World Health Organization classification of lymphoid neoplasms. Blood 2016;127:2375–2390. 26980727 10.1182/blood-2016-01-643569 PMC4874220

[2] Lynch DT Koya S Acharya U . Mantle cell lymphoma. In: StatPearls. Treasure Island (FL): StatPearls Publishing; 2023. 30725670

[3] Fernàndez V Salamero O Espinet B . Genomic and gene expression profiling defines indolent forms of mantle cell lymphoma. Cancer Res 2010;70:1408–1418. 20124476 10.1158/0008-5472.CAN-09-3419

[4] Argatoff LH Connors JM Klasa RJ . Mantle cell lymphoma: a clinicopathologic study of 80 cases. Blood 1997;89:2067–2078. 9058729

[5] Watson S . Combination Chemotherapy for Mantle Cell Lymphoma: WebMD. Accessed May 10, 2023. https://www.webmd.com/cancer/mcl-combination-chemotherapy

[6] Zelenetz AD Abramson JS Advani RH . NCCN clinical practice guidelines in oncology: non-Hodgkin’s lymphomas. J Natnl Comprehens Cancer Netw 2010;8:288–334. 10.6004/jnccn.2010.0021 20202462

[7] Inwards DJ Cilley JC Winter JN . Radioimmunotherapeutic strategies in autologous hematopoietic stem-cell transplantation for malignant lymphoma. Best Pract Res Clin Haematol 2006;19:669–684. 16997176 10.1016/j.beha.2006.05.004

[8] Questions Remain About Best Frontline Treatment in Mantle Cell Lymphoma. Healio. 2022. Accessed 10 May 2023. https://www.healio.com/news/hematology-oncology/20220228/questions-remain-about-best-frontline-treatment-in-mantle-cell-lymphoma

[9] Wen T Wang J Shi Y . Inhibitors targeting Bruton’s tyrosine kinase in cancers: drug development advances. Leukemia 2021;35:312–332. 33122850 10.1038/s41375-020-01072-6 PMC7862069

[10] Woyach JA Furman RR Liu TM . Resistance mechanisms for the Bruton’s tyrosine kinase inhibitor ibrutinib. N Engl J Med 2014;370:2286–2294. 24869598 10.1056/NEJMoa1400029 PMC4144824

[11] FDA grants accelerated approval to pirtobrutinib for relapsed or refractory mantle cell lymphoma. U.S. food and drug administration; Accessed 10 May 2023. https://www.fda.gov/drugs/resources-information-approved-drugs/fda-grants-accelerated-approval-pirtobrutinib-relapsed-or-refractory-mantle-cell-lymphoma#:~:text=On%20January%2027%2C%202023%2C%20the,full%20prescribing%20information%20for%20Jaypirca

[12] Jensen JL Mato AR Pena C . The potential of pirtobrutinib in multiple B-cell malignancies. Ther Adv Hematol 2022;13:20406207221101697. 35747462 10.1177/20406207221101697 PMC9210100

[13] Mato AR Shah NN Jurczak W . Pirtobrutinib in relapsed or refractory B-cell malignancies (BRUIN): a phase 1/2 study. Lancet 2021;397:892–901. 33676628 10.1016/S0140-6736(21)00224-5 PMC11758240

[14] John P Cunha D , FACOEP. JAYPIRCA SIDE EFFECTS CENTER: RxList; 2023. Accessed 3 August 2023. https://www.rxlist.com/jaypirca-side-effects-drug-center.htm

[15] Ito R Eyre TA Shah NN . MCL-135 BRUIN MCL-321, a Phase 3 Open-Label, Randomized Study of Pirtobrutinib Versus Investigator Choice of BTK Inhibitor in Patients With Previously Treated, BTK Inhibitor Naïve Mantle Cell Lymphoma (Trial in Progress). Clin Lymphoma Myeloma Leuk 2022t;22(Suppl 2):S395–S396.

[16] Wang M Shah NN Alencar AJ . Pirtobrutinib, a next generation, highly selective, non-covalent BTK inhibitor in previously treated mantle cell lymphoma: updated results from the phase I/II BRUIN study. Blood 2021;138(Suppl 1):381.

